# Extension Trial of Qigong for Fibromyalgia: A Quantitative and Qualitative Study

**DOI:** 10.1155/2013/726062

**Published:** 2013-08-28

**Authors:** Jana Sawynok, Mary Lynch, Dana Marcon

**Affiliations:** ^1^Department of Pharmacology, Dalhousie University, 5850 College Street, P.O. Box 15000, Halifax, NS, Canada B3H 4R2; ^2^Departments of Anesthesiology, Psychiatry and Pharmacology, Dalhousie University, QEII Health Sciences Centre, Dickson Centre, 5820 University Avenue, Halifax, NS, Canada; ^3^Personal Training Clinic, Halifax, NS, Canada

## Abstract

This extension trial is an open-label observational trial of 20 subjects with fibromyalgia who undertook level 2 Chaoyi Fanhuan Qigong (CFQ) training following an earlier controlled trial of level 1 CFQ. Subjects practiced 60 min/day for 8 weeks and continued some daily practice for 6 months. Quantitative measures, assessed at baseline, 8 weeks, 4 and 6 months, were of pain, impact, sleep, physical and mental functions, and practice time. Qualitative comments also were recorded. Compared to baselines, CFQ practice led to significant improvements in pain, impact, sleep, and physical function in the 13 subjects (65%) who completed the trial; changes were present at 8 weeks and were maintained for the 6-month trial duration. A highly motivated subgroup of *N* = 5, who practiced the most, had the best outcomes in terms of end symptomology, and qualitative comments indicated health benefits in other domains as well. Qualitative comments by the remaining *N* = 8 trial completers and *N* = 7 withdrawals indicate different experiences with the practice. This extension trial indicates that diligent CFQ practice over time produces significant health gains in fibromyalgia in a subset of individuals. Future studies will need to address factors that might predispose to favourable outcomes.

## 1. Introduction

Qigong, which means the cultivation of *qi *or the energetic essence of the human being, has a long history extending thousands of years, is part of contemporary Traditional Chinese Medicine and constitutes a distinct approach to healing [1, 2]. Qigong practice (internal qigong) involves physical movements and postures, breathing practices, and meditative techniques, and there are many forms [1, 2]. Multiple forms of qigong are now practiced in many countries, and there is an emerging literature reporting health benefits in several chronic health conditions [3, 4]. More recently, qigong has been characterized as “mindful exercise” [5] or “meditative movement” [6] and this provides domains in which components of the practice can be considered.

Fibromyalgia is a chronic pain condition associated with sleep and mood disturbances and diminished quality of life [7, 8]. In 2008, a Swedish study reported long-term benefits in pain, sleep, and psychological function in fibromyalgia in a controlled study involving daily practice of qigong for 7 weeks [9]. In Nova Scotia, following completion of a pilot trial involving a similar regimen [10], we conducted a controlled trial of Chaoyi Fanhuan Qigong (CFQ) [11] for fibromyalgia in which subjects were trained in level 1 CFQ movements, practiced daily for 8 weeks, and were encouraged to continue practice for 6 months [12]. Wait list subjects served as a control group and underwent instruction and practice at the end of the wait time. In both cohorts (immediate, delayed), there were significant improvements in pain, impact, sleep, physical function, and mental function, and benefits were maintained to 6 months [12]. Following that trial, some subjects (*N* = 10) voluntarily undertook level 2 CFQ training (meditation) in the community, continued their practice, and were known, anecdotally, to be experiencing further health improvements. Furthermore, cases of marked benefits in fibromyalgia in those undertaking community-based training and engaging in extensive CFQ practice were documented [13]. In view of this, we undertook an extension trial in which participants who completed the controlled trial were invited to participate in a further trial in which level 2 CFQ (meditation) was added to the level 1 CFQ (movement) instruction. The goals of the extension trial were (a) to determine whether level 2 CFQ instruction and further practice would produce additional benefits in fibromyalgia and (b) to document health effects of extensive practice of CFQ. Fibromyalgia is a complex and difficult condition to treat [14] and is a challenge for both patients and clinicians [15]. While drugs are approved for treating fibromyalgia, these have limited efficacy [7, 14, 16]. Furthermore, multimodal treatments show limited effectiveness in the long-term [17], and longitudinal benefits of treatments are generally modest [18]. It is therefore important to explore all possible modalities for this condition, including practices considered as complementary and alternative medicine or CAM [19, 20]. 

## 2. Methods 

### 2.1. Participants

The controlled trial of level 1 CFQ was conducted between September 2009 and July 2011 [12]. The current extension trial took place between March and September 2012 and was conducted at the Pain Management Unit, Queen Elizabeth II Health Sciences Centre, Halifax, with Ethics Review Committee approval. Original participants were invited to a review session of trial results (25 attended) and invited to join the extension trial. *N* = 20 entered the 6-month open-label extension phase. Participants were assigned the same study number as in the original trial for longitudinal tracking. During the initial trial, there were several training cohorts, so the amount of time between level 1 and level 2 CFQ instruction was variable.

### 2.2. Training and Practice

Extension trial participants received training in level 2 CFQ by a certified instructor (DM) at two half-day (4 hours each) training sessions. This was followed by weekly group practice sessions (60 mins) for 8 weeks. Participants were required to practice daily for 60 mins during these 8 weeks; following that, continued daily practice was encouraged to the end of the trial. 

CFQ was developed in the 1990s [11] and is available locally in Nova Scotia. Level 1 instruction consists of a set of 7 movements which are slow and rhythmical and are accompanied by a relaxed mental state and connection-to-body feeling. In a set, there are 10 repetitions of movements 1–5, and 5 repetitions of movements 6-7; each set takes 10–12 mins to complete. Level 2 consists of instruction in meditation techniques, primarily sitting meditation, but standing and lying meditation also were presented as practice options. Sitting meditation involves bringing attention to the interface with the chair, feeling the chair, disengaging thought, and switching into open awareness. With standing meditation, attention is brought to the lower abdomen and into the feet, with lying meditation, to the lower abdomen and contact with the floor, with similar feeling-of-contact and mental instructions. Meditation is practiced for 30 min intervals. Participants were required to practice for a total of 60 mins per day, with a recommendation of level 1 CFQ (movements) and level 2 CFQ (meditation) for 30 mins each.

### 2.3. Outcome Measures

Quantitative measures were the same as in the level 1 CFQ controlled trial [12] and comply with recommendations for core domain assessments in chronic pain and fibromyalgia trials [21, 22]. Measures included (1) pain (NRS-PI, 11-point numerical rating scale pain intensity, with anchors of “no pain” and “pain as bad as you can imagine”), (2) Fibromyalgia Impact Questionnaire (FIQ), (3) Pittsburgh Sleep Quality Index (PSQI), and (4) SF-36 Health Survey (physical and mental scores analysed separately). Participants also completed the following: (5) Patient Satisfaction Scale (How satisfied are you with the qigong treatment? with −3 = very unsatisfied, 0 = neither satisfied nor dissatisfied, +3 = completely satisfied) and (6) Patient Global Impression of Change Scale (How would you grade your overall fibromyalgia condition since using qigong? with −3 = very much worse, 0 = no change, +3 = very much improved). They were also asked to indicate the following: (7) daily practice time (categories of 60, 45, 30, 15, 0 min) and (8) side effects from their practice. A qualitative questionnaire, which invited open-ended comments on experiences, also was included. (At Baseline: briefly describe changes to your health with completion of the first phase of the trial. At 8 weeks: describe changes in your health that you attribute to CFQ practice over the past 8 weeks. At 4 and 6 months: describe changes in your health since entering the study that you attribute to CFQ practice.) Those that had previously voluntarily undertaken level 2 CFQ training were identified in this questionnaire. Assessments were completed on the day of training (baseline) and at the last weekly group session (8 weeks); 4–6 months reports were returned by mail.

### 2.4. Statistical Analysis

Statistical analysis was conducted on the group that completed the trial to 6 months, the per protocol group. Baselines were compared with 8-week, 4- and 6-month values. Pain, sleep, and SF-physical comparisons were performed using one-way repeated measures analysis of variance (ANOVA), with the Holm-Sidak method for multiple comparisons versus baseline. FIQ scores failed the equal variance test using this method and were analysed using one-way ANOVA with the Student-Newman-Keuls test for pairwise comparisons. SF-mental scores failed variance (one-way repeated measures ANOVA) and normality (one-way ANOVA) and could not be further analysed. 

## 3. Results

### 3.1. Baseline Characteristics

**Figure 1:**
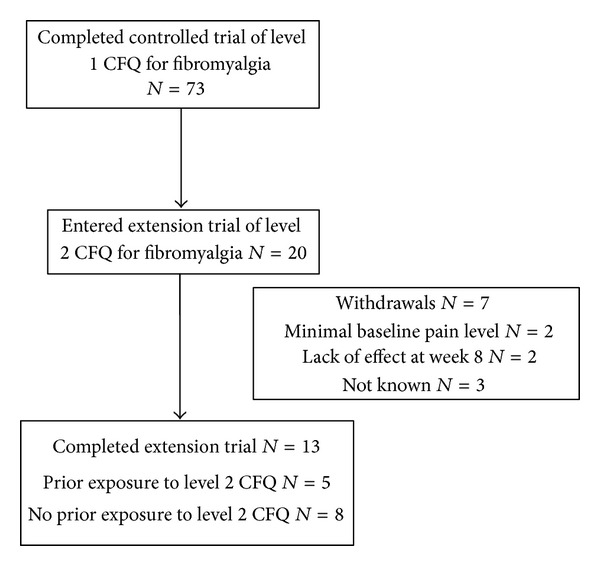
Subject disposition in open-label extension trial of qigong for fibromyalgia.

Demographics and baseline characteristics of trial participants who entered the extension (*N* = 20) were extracted from the earlier trial [12] (Table 1). Their baseline scores for pain, impact, sleep, and physical and mental function indicate that participants are representative of the original groups (pain 6.45, 6.65; impact 65.53, 59.73; sleep 13.79, 12.35; SF-physical 29.94, 33.22, SF-mental 38.13, 39.03 for immediate and delayed training groups, resp.). *N* = 13 (65%) completed the 6-month period; *N* = 5 had previously voluntarily undertaken level 2 CFQ training; *N* = 7 withdrew from the study (4 by week 8, 3 more by 4 months) (Figure 1). Side effects included pain (*N* = 5), cooler body (*N* = 1), headache (*N* = 1), discolored hands and feet (*N* = 1), intermittent cough (*N* = 1), and increased stress (*N* = 1); none of those reporting side effects withdrew.

### 3.2. Quantitative Assessments

**Figure 2:**
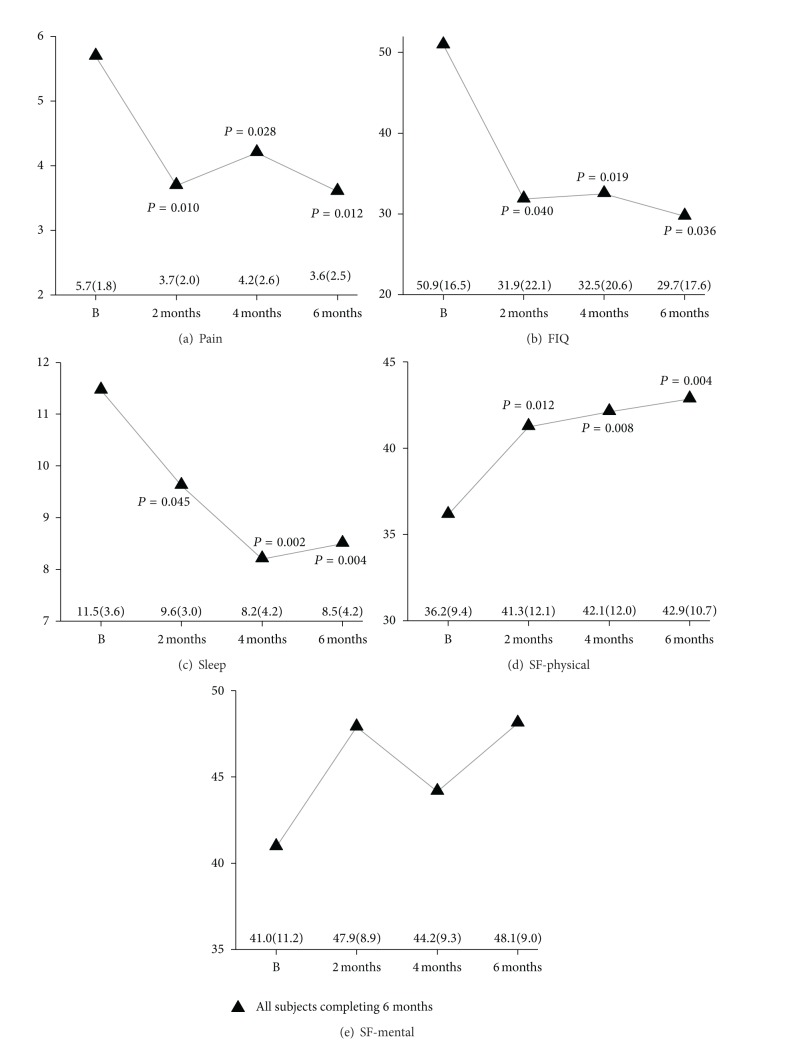
Effects of qigong practice on pain, impact, sleep, and physical and mental functions in all participants who completed the extension trial over 6 months (*N* = 13). (a) Pain (Numerical Rating Scale Pain Intensity), (b) impact (Fibromyalgia Impact Questionnaire), (c) sleep (Pittsburgh Sleep Quality Index), (d) physical function (SF-Health Survey, Physical), and (e) mental function (SF-Health Survey, Mental). Values shown in panels are means; mean (SD) values depicted in the lower panel. *P* values shown for values significantly different (*P* < 0.05) from baseline (B).

**Figure 3:**
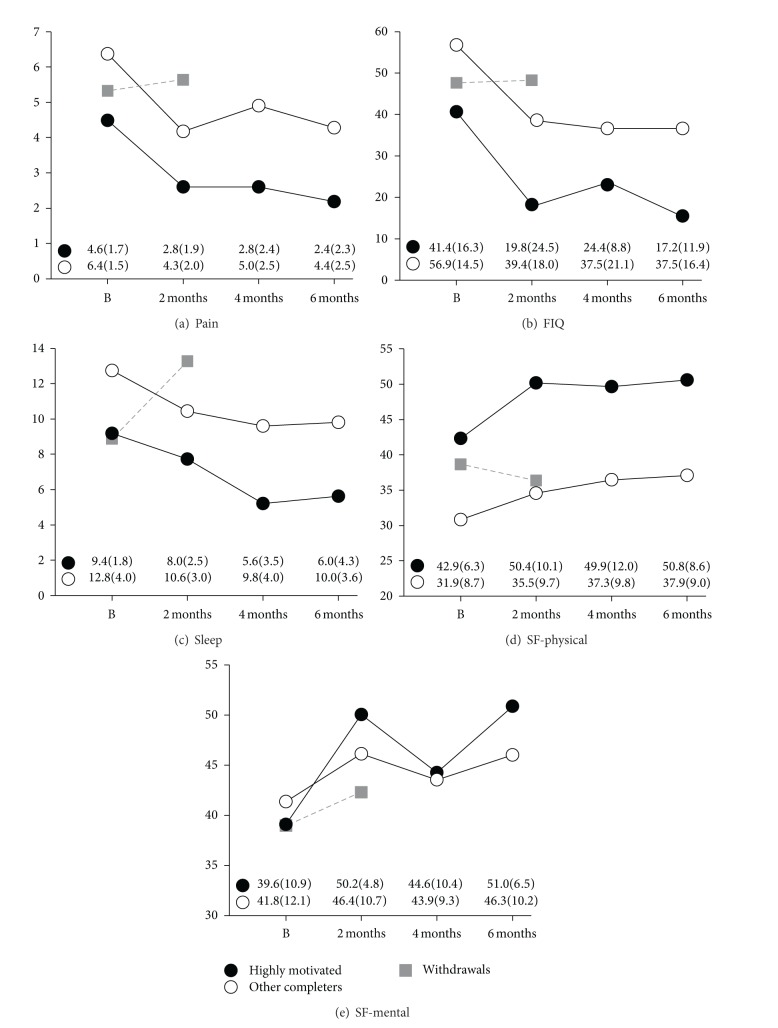
Effects of qigong practice on pain, impact, sleep, and physical and mental functions in subgroups who had previously voluntarily undertaken level 2 CFQ training (*N* = 5, highly motivated) (black circles) and others who completed the 6-month extension trial (*N* = 8) (white circles). (a) Pain (Numerical Rating Scale Pain Intensity), (b) impact (Fibromyalgia Impact Questionnaire), (c) sleep (Pittsburgh Sleep Quality Index), (d) physical function (SF-Health Survey, Physical), and (e) mental function (SF-Health Survey, Mental). Values shown are means; mean (SD) values depicted in the lower panel. Mean values for those who withdrew from the trial are depicted by grey squares (*N* = 7 at baseline, *N* = 3 at 8 weeks).

The results for pain, impact, sleep, and physical and mental functions for the entire group are presented in Figure 2. Improvements in quantitative scores were generally manifested by 8 weeks and maintained at 4–6 months. There were significant pre- versus postpractice effects for pain, impact, sleep, and physical function. Mental function showed directional trends towards improvement (see also qualitative comments). Data was also analysed as subgroups: (1) those who voluntarily undertook level 2 training prior to the extension trial and were highly motivated (*N* = 5), (2) other participants who completed the trial (*N* = 8), and (3) those that withdrew from the trial (*N* = 7). These results are presented in Figure 3. Both subgroups completing the trial showed improvements in pain, impact, sleep, and physical and mental functions, with similar pre-post practice differences. Baseline values appeared different between subgroups, with the highly motivated subgroup showing less severe symptoms, but only physical function was significantly different (*P* = 0.04, Student's *t*-test). 

Self-reported practice times from the categorical checklist are indicated in Table 2. On average, the group completing the trial practiced 35–44 min/day throughout. The *N* = 5 highly motivated subgroup complied with 60 mins/day practice over 8 weeks and maintained this over time (66, 57, and 60 mins/day); the remaining subgroup completing the trial had lower practice times at 8 weeks and these declined over time (38, 24, and 20 mins/day). Those that withdrew from the trial had the lowest practice times at 8 weeks.

Patient satisfaction scores (mean ± SD) for those completing the trial were uniformly high at all intervals (7 indicates “completely satisfied”): 5.9 ± 1.8 at 8 weeks, 5.8 ± 1.9 at 4 months, and 6.3 ± 1.0 at 6 months. Patient global impression of change scores (mean ± SD) also were uniformly high (7 indicates “very much improved”): 5.6 ± 1.6 at 8 weeks, 5.7 ± 1.4 at 4 months, and 5.6 ± 1.0 at 6 months. No subgroup analysis of these scores was undertaken.

For those who completed the trial (*N* = 13), the number of pain medications (mean ± SD) at entry was 3.2 ± 1.3; at the end of the trial, this was 1.1 ± 1.3. Six participants (46%) reported no longer taking any pain medications at the end of the trial; all had been taking pain medications at the beginning of the trial.

### 3.3. Qualitative Assessments

Table 3 presents qualitative comments by the *N* = 5 who voluntarily undertook level 2 CFQ training prior to entering the extension trial. Baseline comments reflect experiences following the previous trial, as well as subsequent voluntary practice. Improvements in many areas were identified after 8 weeks and consolidated at 4 and 6 months. Comments indicate further health benefits beyond those documented by quantitative results. Thus, there are reports of improvements in asthma (006, 007), food allergies (006, 007), allergies and sinus headaches (098), chemical sensitivities (006, 007), carpel tunnel symptoms, tendonitis (006), migraine headaches (006), weight loss (036), vision (036), blood pressure (087), and mood (087). In several instances, there was discontinuation of medications for asthma (006, 007), sleep (006), mood (007), and pain (006, 007). In some cases, multiple drugs were discontinued (006, 036). There are many comments relating to marked improvements in quality of life (006, 007, 036, 087, 098). Some participants indicate substantial amounts of practice in this open-ended format (10–15 hrs/week) (006, 007, 036).

Table 4 summarizes qualitative comments offered by remaining participants who completed the extension trial (*N* = 8). Several comments are positive and, in addition to mentioning symptoms evaluated in quantitative results, there is mention of improvements in blood pressure (029) and muscular dystrophy symptoms (048). There are also comments indicating pain as a result of practice (019, 029, 044, 075) (see also side effects) and difficulties with meditation (019, 029, 042). Self-reported practice times in the open format for this group are lower than those in the highly motivated group. 

Table 5 summarizes qualitative comments by those who withdrew from the trial. There are several positive comments as a result of earlier qigong experiences (004, 005), including reduced blood pressure (004, 087), stopping medications (025, 082), and quality of life improvements (089). There are also challenges with the meditation instruction (060). Two who withdrew had low baseline levels of pain (2-3) (004, 005) and would not have been included in the trial if a minimum symptom severity (≥4) was required.

## 4. Discussion

This was an extension to a previous randomized controlled trial in which level 2 CFQ (meditation) was added to level 1 CFQ (movement) for subjects with fibromyalgia. Qigong was recently characterized as meditative movement [6], and we were interested in determining whether components of the practice could be examined sequentially. We also wished to document health effects in those who engaged in extensive qigong practice. The trial is best characterized as an open-label observational trial; it is also a long-term trial (2-3 years) and provides valuable longitudinal information on the effects of diligent practice. Quantitative results in those who completed the extension trial indicate significant improvements in core domains of fibromyalgia as a result of qigong practice. Subgroup analysis indicates that the highly motivated group and others who completed the trial attained similar reductions in pain, impact, and sleep impairment, and improvements in function. Of note in the highly motivated subgroup is the observation that postpractice absolute values for pain (scores of 2-3) and impact (scores of 15–25) suggest mild symptomology. Qualitative comments by the highly motivated subgroup recapitulate benefits in core domains, as well as indicating diverse further health benefits (improvements in allergies/sensitivities, migraines, asthma, blood pressure, and vision). Benefits include resumption of exercise and weight loss, which further contribute to improved health. Qualitative comments for others who completed the trial also contain positive health comments but are more moderate in tone. Subgroups were distinguished in terms of self-reported qigong practice times (highly motivated > completers > withdrawals). 

Collectively, these quantitative and qualitative results indicate that dedicated CFQ practice over time produces marked and sustained benefits in core domains relevant to fibromyalgia, as well as additional health benefits. These outcomes are generally supported by other trials of fibromyalgia and related conditions, where sustained qigong practice (daily for 6–12 weeks) is involved [9, 23, 24]. Other trials of qigong for fibromyalgia used qigong as part of a weekly regimen over 8–12 weeks and reported more equivocal results [25–27]. Improvements in some conditions in the present trial noted in qualitative comments (e.g., blood pressure) are supported by an emerging literature [28]. Of particular note is the magnitude of change in several conditions (asthma resolving, improved mobility, weight loss, improved eyesight, resolution of carpel tunnel symptoms, and resolution of allergies/sensitivities). Beneficial effects were most prominent in the group that engaged in the most qigong practice. Similar marked improvements in diverse conditions were observed after extended CFQ practice in case reports [13]. Based on these results, and recognizing that these benefits occur in those who have been compromised for an extended period of time (fibromyalgia mean duration 11.8 years, *N* = 13), further studies on the health benefits of qigong practice, and especially extended practice, in fibromyalgia as well as other chronic health conditions, are encouraged. In some frameworks, fibromyalgia is considered a central sensitization syndrome along with other disorders (e.g., chronic fatigue syndrome, regional pain disorders, irritable bowel syndrome, and headache disorders) [29, 30], and it would be interesting to ascertain effects of qigong in these conditions. Thus, it was recently reported that qigong is of benefit in chronic fatigue syndrome [24].

There are several methodological issues to consider in relation to this extension trial. (1) *Qigong, a Complex Practice.* Qigong, along with tai chi, is characterized as meditative movement [6] and is a complex intervention with many components potentially contributing to efficacy [3, 5, 31]. It is impossible to blind qigong practice, and trial designs such as randomized controlled trials of fixed protocols, community-based observational trials, cross-sectional studies of long-term practitioners, and studies that integrate qualitative methods to capture the richness of participant experiences have been encouraged [32]. The current extension trial incorporates several of these recommended trial design elements. (2)* Plurality of Forms, Components. *There are many forms of qigong [1, 2], and the contribution of a particular component of activity to the outcome is not clear. Considering qigong as meditative movement [6] is useful as it provides domains for comparison, and future trials can compare qigong with exercise regimens or with meditation groups. However, there are various forms (aerobic, strength, and flexibility) and intensities (mild, moderate) of exercise, and this area has its own research challenges [33, 34]. Additionally, there is a multiplicity of mind-body interventions (mindfulness, meditation) and this field also contends with methodological challenges [35]. The current trial did not have a comparison group and assessed pre- versus post-intervention outcomes in a quantitative and qualitative manner; it is pragmatic and akin to clinical practice. (3) *Extent of Practice, Effectiveness of Practice.* How much qigong practice is needed for clinically meaningful effects (“minimal dose”), and whether further practice leads to better outcomes (“dose-response relationship”), are important issues. Furthermore, there is the issue of effectiveness (“bioavailability”), of whether all time spent seemingly engaged in an activity is equivalent [32]. *Post hoc* analysis of outcomes in relation to extent of practice indicated differences between those who practiced per protocol or minimally in the earlier controlled trial [12]. In the present trial, we also observed a practice-response relationship, as those who practiced the most had the best outcomes. Systematic assessment of practice time is feasible using standard protocols in controlled trials, as well as with observational trials, and is encouraged for all qigong trials. Furthermore, meta-analysis of qigong trials for fibromyalgia will need to consider this factor in clustering studies for analysis [19, 36]. Finally, motivation for or barriers to continued/extensive practice (e.g., use of booster sessions, group versus individual sessions, nature of instruction, and effectiveness of different instructors) will need to be considered in future studies. (4) *Identifying Those Who Benefit from Qigong.* In this trial, 13/20 (65%) undertook additional instruction, completed 6 months, and experienced significant pre- versus postpractice benefits. This subgroup represents 18% of original trial completers (13/73). Extensive qigong practice requires a considerable commitment and will not be suitable for everyone for many reasons. However, because multiple health benefits can result from diligent practice, as documented here and in case studies of CFQ [13], there will be some willing to engage in such practice if it means relief from a long-standing condition which has not been amenable to other approaches. Future studies on qigong could include additional assessments of participant factors (e.g., psychosocial profile, locus of control, and attitudes towards CAM), to determine those that might predispose to favourable outcomes. Thus, those with fibromyalgia exhibit differences in health related locus of control, cognitive attributes, and perceived social support compared to other chronic pain populations and/or controls, for example, [37, 38]. This approach fits within the conceptual frameworks of attribute-treatment interactions [39], preference trials [40], and personalized medicine.

## 5. Conclusion

This open-label extension trial indicates that diligent practice of CFQ, a particular form of qigong, produces sustained benefits in fibromyalgia as indicated by quantitative assessments in core domains for fibromyalgia. Qualitative comments indicate health benefits in other areas as well. Benefit is related to extent of practice.

## Figures and Tables

**Table 1:** Demographics and other characteristics of trial participants entering the extension trial (extracted from information provided at entry to the earlier controlled trial) [12].

Variable	Extension trial group (*N* = 20)
Gender F : M	20 : 0
Age at enrollment, years (SD)	53 (9.3)
Duration of fibromyalgia, years (SD)	11.5 (6.7)
BMI at enrollment (IQR)	31.3 (7.8)
Pain medications, mean number (SD)	3.0 (1.6)
Other medications, mean number (SD)	5.2 (2.9)
Baseline NRS-PI, mean (SD)	6.5 (1.3)
Baseline FIQ, mean (SD)	58.8 (15.8)
Baseline PSQI, mean (SD)	14.0 (3.1)
Baseline SF-physical, mean (SD)	31.5 (10.7)
Baseline SF-mental, mean (SD)	41.8 (11.3)

**Table 2:** Self-reported qigong practice times (mean ± SD, combined level 1 and level 2 CFQ) for extension trial participants as indicated in the categorical checklist (0, 15, 30, 45, 60 min options).

	8 weeks (min/day)	4 months (min/day)	6 months (min/day)
*N* = 13 trial completers	44 ± 21	38 ± 23	35 ± 26
*N* = 5 motivated	66 ± 13	57 ± 20	60 ± 15
*N* = 8 others	38 ± 14	24 ± 13	20 ± 13
*N* = 7 withdrawals	20 ± 10	—	—

**Table 3:** Qualitative comments by *N* = 5 subjects who voluntarily undertook level 2 CFQ training after the level 1 trial and then completed the extension trial. Baseline comments relate to experiences following the trial and then community-based instruction and continued practice. Comments retain original wording and content but are edited to remove identifying and extraneous information. Original trial number, age, and duration of fibromyalgia (FM) are indicated for each subject (mo = month).

Subject	Baseline comments	Comments following qigong practice
*1 (006)* Age: 55 FM: 7 years	Less pain, more relaxed, slept better, increased energy. [With level 2] when practice diligently, experience calmness, less pain, better sleep; when skip practice, am angry, tired, and [have] major migraines.	Week 8: food allergies have greatly improved. No longer have asthma and am off puffers; have been able to increase exercise without shortness of breath. Wood smoke no longer bothers me. Sleep less and have more energy, better quality of sleep. Am calmer. Carpal tunnel [symptoms] and tendonitis are gone. Mind is less “brain fogged.” Much less pain and stiffness; rare migraines versus frequent migraines. Month 4–6: have discontinued inhalers, no indication of asthma. Am exercising more (biking 20 kms/day). Sleep well and feel well. Food allergies are nonexistent and chemical sensitivities much reduced (4 mo). Have discontinued all prescription medications (Ventolin, Elavil, Tryptophan, Advil, Imitrex). Am able to do things I haven't done in years, have more energy (6 mo). *Note: reports practicing 10–14, then 14–18 hrs per week *

*2 (007)* Age: 25 FM: 11 years	Allergies are much better, pain is reduced, much happier and more at peace. Can deal with stress better. [With level 2] quality of sleep has improved, pain reduced further.	Week 8: function very well on less sleep. Food allergies are improved. Asthma has improved and am no longer on puffers. Have started running and hiking. Pollen season had no effect on me this year; usually have severe pollen allergies. Was allergic to grass [asthma attack and /or headache]; this year can smell cut grass [with no symptoms] and it smells good. Better quality of sleep, rarely wake at night. Mind is clearer, am more alert. Am in less pain and have gone a very long time without needing Tylenol. Month 4–6: off all sleep medications (Tryptophan, Elavil), and quality of sleep has improved. Bike 1 hr (20 kms) every weekday morning, something I couldn't do before. Food allergies have improved, [now] regularly eat cheese or yogurt [strong allergies before]. Other “allergies” (scents) no longer bother me (4 mo). Asthma no longer an issue (6 mo). *Note: reports practicing 10, then 14–15 hrs per week *

*3 (036)* Age: 57 FM: 10 years	At the beginning was using a walker and then a cane; now walk without a cane. Was depressed and felt hopeless about my health, now feel grounded and peaceful. Have reduced amount of medication by half. Have lost 135 pounds on my journey with the practice as well. [With level 2] greater personal peace.	Week 8: Qigong has changed my life in so many positive ways. Have learned to accept pain. Am much more accepting and much calmer. When I started Qigong, I was using a walker, then canes, and now I am able to walk without canes. Have also lost a lot of weight which I hadn't been able to lose before. Feel at home in my body. The benefits of Qigong are many. Month 4–6: [No comments at 4 mo] Qigong has given me my life back. When I started, was using a walker, couldn't sleep and was in terrible pain. Now feel peaceful; am walking, sleeping well; pain levels have come down considerably. Eyesight has come down from 6.25 & 6.75 to 4.25 and 4.50. Was housebound, now I can walk the dog and go out to do errands. Still have problems with fatigue but overall, am much better (6 mo). *Note 1: reports practicing 12, then 7–12 hrs per week* *Note 2: 036 also offered written information on experiences with CFQ prior to entering the extension trial. Before the earlier trial, she was practically housebound and had little hope for improvement. She had been diagnosed with fibromyalgia, psoriatic arthritis, bursitis, asthma, high blood pressure, irritable bowel syndrome, temperomandibular joint disorder, depression, and severe sleep apnea. She was taking 4 pain medications (codeine contin, pregabalin, tramadol/acetaminophen, and hydromorphone) as well as 7 other medications. By the time she entered the extension trial, her experiences had allowed her to discontinue hydromorphone, tramadol/acetaminophen, methotrexate, flovent, salbutamol, and her codeine contin intake had dropped dramatically. She also no longer needed her cane or CPAP (continuous positive airway pressure) machine. She had lost a substantial amount of weight. Her case is interesting, as she started classes with “little hope that it would help. How could these simple movement patterns help?” *

*4 (087)* Age: 46 FM: 25 years	Biggest thing is mood; from attempting suicide to having hope. Mobility much better, less pain. Able to tolerate things. Like a weight has been lifted off my shoulders. [With level 2] deeper understanding of energy flows, blockages which cause pain. Ability (sometimes) to let the pain come and focus on something else.	Week 8: mood elevated. Sugars much better controlled. Blood pressure is excellent. Less flares of fibromyalgia. Better flexibility/mobility, especially with arms and shoulders. Lots of pain after doing Qigong, but it is just memories working through. Have had black hands and feet, dizziness and bruises that appear for no reason. Realistic expectations with health, especially with my diabetes. Have pain and tired all the time, but live as best I can. Qigong keeps my mood and perspective even. Month 4–6: mood is the huge change from attempting suicide to going [on a trip] and planning more travel. Enjoy my work and am more tolerant of others. Body is a lot more flexible and mobile, get out walking a few kms most days. Am doing things that I never thought I would be able to do again (4 mo). Always mood much better after any amount of Qigong. Mobility greatly increased (6 mo). *Note: reports practicing 4, then 5 hrs per week *

*5 (098)* Age: 54 FM: 18 years	Improved sleep, decreased pain, more calm and peaceful. [With level 2] allergies improved, fewer sinus headaches and infections. Happier and deal with stressful situations with increased calmness.	Week 8: fewer sinus headaches due to allergies. Restorative sleep more frequent. More positive about life and its challenges; more peaceful, tolerant and understanding. Cut back in the number of hours spent at work. Not wasting energy on guilt and worry over things I cannot control. Month 4–6: decrease in allergies and sinus problems. Decrease in tolerance to stress due to uncovering underlying anxiety; expect this to change again as soon as I continue my practice. Overall condition has improved (4 mo). Ability to cope with stress has increased. Improvement in severity of pain and frequency (6 mo). *Note: reports practicing 6, then 4-5 hrs per week *

**Table 4:** Qualitative comments of the remaining *N* = 8 subjects who completed the extension trial. Baseline comments relate to experiences following level 1 CFQ. Comments retain original wording and content but are edited to remove identifying and extraneous information. Original trial number, age, and duration of fibromyalgia (FM) are indicated for each subject (mo = month).

Subject	Baseline comments	Comments following qigong practice
*1 (019)* Age: 59 FM: 14 years	Qigong helped me enormously at first, physically and emotionally. Have not practiced faithfully; do about 15 mins/day. Most negative health changes are due to arthritic changes, degenerative discs, pinched nerve in neck, bad shoulder, foot, and hand.	Week 8: for the first 2 weeks felt worse, or not improved; had a lot of movements during meditation which I found exhausting, woke stiff and sore. By week 3/4 was feeling a bit more energetic and woke feeling better, but tired. By week 6, was feeling good upon waking. By week 7/8, am feeling good most of the day. Back gets sore by late afternoon. Burning and stiffness due to fibromyalgia are mostly gone. Have more energy, less pain. Month 4–6: had to stop sitting meditation as it caused lower back pain, so do lying meditation; very relaxing, fall asleep within minutes. Sleep more soundly and have more energy. Less fibromyalgia pain (still have arthritis aches—spine and hand) (4 mo). Am more relaxed, fall asleep more easily. *Note: reports practicing 5, then 2-3 hrs per week *

*2 (029)* Age: 65 FM: 9 years	Weight loss, tightening of core and leg muscles, walk further. Blood pressure decreased [154 to 110], off meds for over a year. Overall healthier. Immune system has improved.	Week 8: there haven't been many changes. Stopped doing meditation as found it hurtful (physically) and depressing (mentally). Month 4–6: bad fall and having trouble healing. Stopped Qigong due to injury. *Note: reports practicing 4, then 0–2 hrs per week *

*3 (042)* Age: 44 FM: 12 years	Because of disability, live in poverty with limited resources. Stressful housing situations have created health issues. CFQ has gotten me through crises. When not in crisis, very noticeable positive effects.	Week 8: anxiety up and down. Level 2 caused temporary overwhelming emotional responses; however, they passed. Less muscle tension and tightness. Month 4–6: negative changes in health since learning CFQ—central nervous retinopathy, psoriasis, and high cholesterol. Positive changes—less pain, headaches, stiffness, aches, increased mobility, and sleep. *Note: reports practicing 5, then 3-4 hrs per week *

*4 (043)* Age: 46 FM: 1 year	Understand my condition more and therefore am more in control.	Week 8: have learned to control anxiety, am more in control and relaxed. Pain seems to improve after practicing Qigong; it helps me cope with pain. Month 4–6: no changes (4 mo). Have not practiced Qigong (6 mo). *Note: reports practicing 3, then 0-1 hr per week *

*5 (044)* Age: 55 FM: 2 years	[Have] osteoarthritis of back and knees and generalized over body, in both thumbs, ankle, elbows; have high blood pressure.	Week 8: feel relaxed; meditation makes me calm and peaceful. Month 4–6: osteoarthritis in left knee and spinal stenosis have worsened [with] certain parts of Qigong practice (4 mo). Meditation gives me peace of mind (6 mo). *Note: reports practicing 3.5, then 1–4 hrs per week *

*6 (048)* Age: 61 FM: 17 years	Qigong has been of great benefit. Because of Muscular Dystrophy, (MD), am limited in moves and practice. Even though am getting weaker due to MD, Qigong has definitely helped with stiffness and stamina.	Week 8: am definitely more flexible and can raise my arms up further and for a longer period of time. The meditation aspect has presented challenges. Month 4–6: even though have not been physically able to practice more than approximately 60 mins/day, it has been of some benefit to me. Hands and feet are warmer, feel calmer. Have recently experienced further progression of MD which has limited me (4 mo). Joints are definitely not as stiff when practicing Qigong (6 mo). *Note: reports practicing 5, then 1.5–3 hrs per week *

*7 (075)* Age: 54 FM: 5 years	Sleep improved, pain decreased, calmer, clearer mind.	Week 8: pain level has increased since starting meditation. Am mostly relaxed during the lying down meditation; pain dissipates during this time. Overall pain has increased, interfering with sleep. Stiffness increased also. Month 4–6: pain levels went up while practicing Level 2. Reduced the amount of time in the last 3 weeks to get a break. Have started the daily routine again to see if there is any improvement (4 mo) Stopped doing meditation and pain levels went down. Still practicing [movements] but not as much as previously. Removed wheat from diet, and stiffness, pain levels and swelling reduced. *Note: reports practicing 6, then 4-5 hrs per week *

*8 (091)* Age: 67 FM: 22 years	Felt better, less pain, more sleep.	Week 8: more relaxed, and joints feel better. Pain seems to be better most of the time. Qigong has helped me cope better. Month 4–6: helped me become more active, given me strength to do more [short walks, biking for first time in years]. Could do more, but my arthritic foot stops me. Qigong has not seemed to help this area. *Note: reports practicing 3, then 2–7 hrs per week *

**Table 5:** Qualitative comments of the *N* = 7 subjects who withdrew from the extension trial. Baseline comments relate to experiences following level 1 CFQ. Comments retain original wording and content and are edited to remove identifying and extraneous information. Original trial number, age, and duration of fibromyalgia (FM) are indicated for each subject.

Subject	Baseline comments	Comments following qigong practice
*1 (004)* Age: 45 FM: 11	Am feeling and doing much better, more happy, more relaxed, more carefree.	Week 8: more energetic, less pain, happier; blood pressure has gone down. Month 4: WITHDRAWAL *Note 1: reports practicing 2 hrs per wk at week 8* *Note 2: reports pain level 2 at baseline, and 3 at week 8 *

*2 (005)* Age: 60 FM: 17	Lowered pain and tiredness, improved mobility and [activities].	Week 8: WITHDRAWAL *Note: pain level 3 at baseline *

*3 (025)* Age: 46 FM: 12	Same as when started except for taking medications (none).	Week 8: WITHDRAWAL

*4 (060)* Age: 54 FM: 18	Improved sleep, but has deteriorated since trial ended. Improved sense of well-being. Increased sense of control of my pain even though pain the same as before.	Week 8: although had a marked improvement in the first level trial, have had no positive response from this second level. Had great difficulty with the training presentation—the material was presented in ways that challenge my worldview. Month 4: WITHDRAWAL *Note: reports practicing 2 hrs per wk at week 8 *

*5 (063)* Age: 53 FM: 10	Pain reduced, better sleep. Diagnosis of diabetes, so was a confusing time.	Week 8: WITHDRAWAL

*6 (082)* Age: 61 FM: 1	Qigong helped me relax. Stopped taking too many pain pills because Qigong is helping.	Week 8: WITHDRAWAL

*7 (089)* Age: 53 FM: 8	Now know the connection between relaxation and how we perceive pain. Qigong makes me feel rested and calm.	Week 8: qigong relaxes me and improves my peace of mind. Am less anxious and less depressed. Can work pain free for longer periods of time. Found the movements beneficial for lower back problems. Find it hard to put time aside [for Qigong], would benefit from longer practice sessions. It has to become a way of life to get full benefits. Month 4: WITHDRAWAL *Note: reports practicing 5 hrs per week at week 8 *
